# First person – Trinitee Oliver

**DOI:** 10.1242/dmm.050850

**Published:** 2024-05-21

## Abstract

First Person is a series of interviews with the first authors of a selection of papers published in Disease Models & Mechanisms, helping researchers promote themselves alongside their papers. Trinitee Oliver is first author on ‘ The glucocorticoid receptor acts locally to protect dystrophic muscle and heart during disease’, published in DMM. Trinitee conducted the research described in this article while a student researcher in Dr Christopher Heier's lab at Children's National Hospital, Washington, DC, USA. She is now a first-year PhD student at the Graduate School of Biomedical Sciences, Cedars-Sinai Medical Center, West Hollywood, CA, USA, investigating sex differences, immunogenetics and precision medicine.


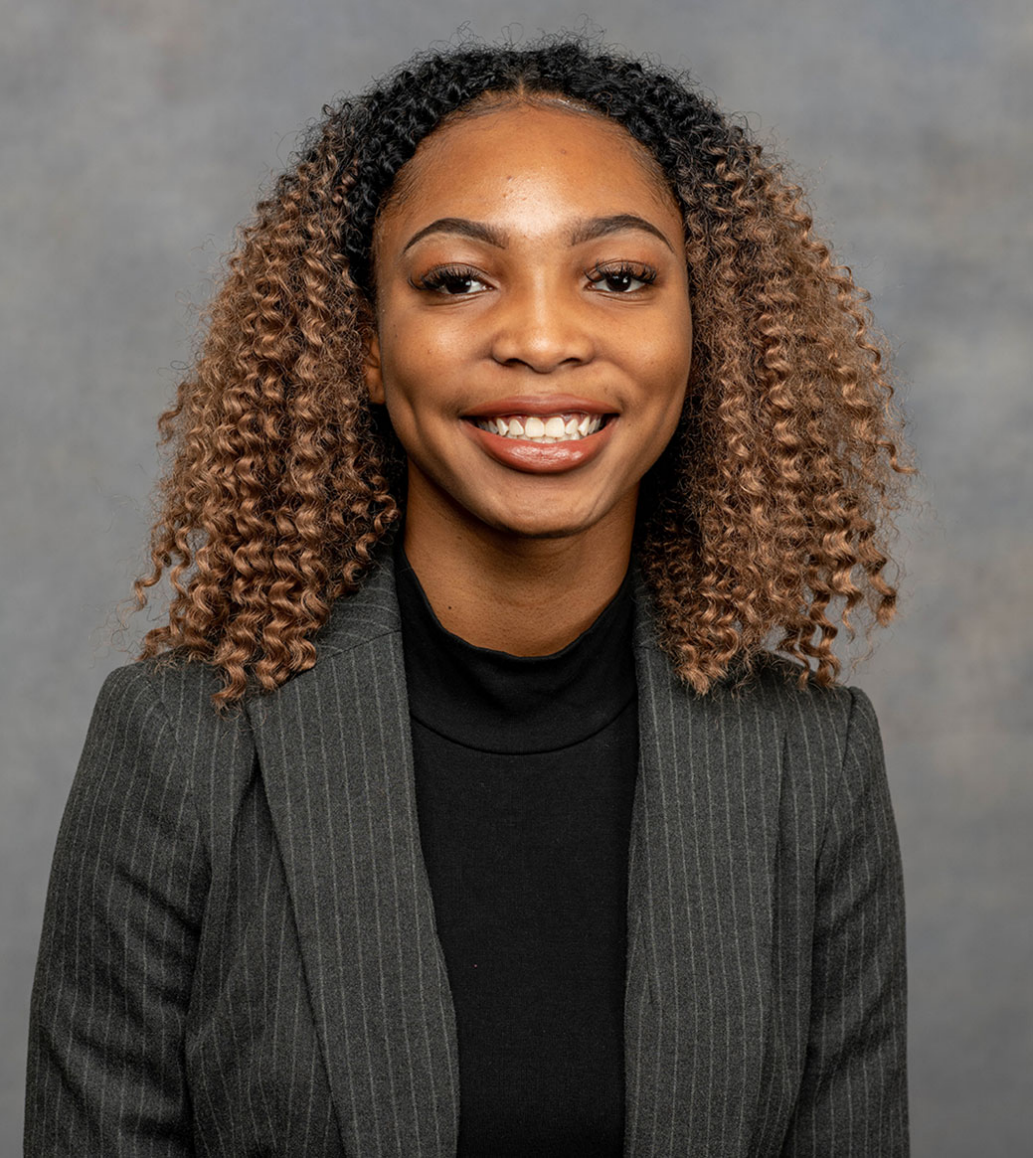


**Trinitee Oliver**

**Who or what inspired you to become a scientist?**

In high school, I shadowed a physician-scientist practicing rheumatology. Throughout the day, I noticed all the patients were women. He then explained to me that clinically we know that females are more likely to have autoimmune diseases, but researchers are still exploring the reasoning behind this. It was the first time I critically thought about sex differences in disease. In college, I studied the role of sex and sex hormones in metabolic, immune and reproductive systems. These experiences brought me to the Heier lab, where I studied Duchenne muscular dystrophy (DMD), an X-linked disorder that primarily affects young male patients.

**What is the main question or challenge in disease biology you are addressing in this paper? How did you go about investigating your question or challenge?**

Dystrophin is the largest gene identified in humans. When someone lacks dystrophin, they experience dystrophy (or the wasting away of a tissue or organ). DMD is a debilitating genetic disorder that causes chronic inflammation and muscle weakness and degeneration. For a long time, the standard of treatment has been prednisone. Prednisone at its best can minimize inflammation and promote an environment for muscle repair; however, at its worst, it can lead to stunted growth and fragile bone. This is particularly concerning for a pediatric-onset disease. We explored the efficacy of the treatment by knocking out the glucocorticoid drug receptor (GR) in the myofibers and cardiomyocytes of mice. We analyzed the protein (by capillary western immunoassay), immune activity (by enzyme-linked immunosorbent assay or ELISA), phenotype (by measuring grip strength), gene expression (by quantitative PCR), heart (by echocardiography), muscle size and pathology (by immunofluorescence) to obtain thorough answers of the role of the GR in mice lacking dystrophin.

This [study] suggests that the body's own corticosteroids actually protect against some of Duchenne muscular dystrophy's bad effects. This discovery opens up new possibilities for treatments that could avoid the nasty side effects of current medicines.

**How would you explain the main findings of your paper to non-scientific family and friends?**

Think of your body like a machine that needs a specific part called dystrophin to keep your muscles strong and your heart healthy. In DMD, this part is missing, leading to serious problems. Doctors often use strong medicines called corticosteroids to help, but these can cause bad side effects. We were curious about whether our body's natural version of these medicines could help or harm people with DMD. In a study, we experimented with special mice that lacked dystrophin, similar to humans with DMD. We also removed a part of the mice that responds to the body's corticosteroids. We found that without this response part, the muscle and heart problems of the mice got even worse. This suggests that the body's own corticosteroids actually protect against some of DMD's bad effects. This discovery opens up new possibilities for treatments that could avoid the nasty side effects of current medicines. The study gives hope for better ways to help people with DMD by understanding how our own body's substances can be part of the solution.

**What are the potential implications of these results for disease biology and the possible impact on patients?**

For patients, the implications could be profound. The nuanced glucocorticoid signaling is important in managing DMD and also hints at the possibility of developing targeted therapies that leverage the beneficial aspects of GR activation. The use of selective GR modulators or the development of novel therapeutic agents like vamorolone, which show efficacy in DMD clinical trials with reduced adverse effects, exemplifies how this understanding could translate into improved clinical outcomes. These therapies could potentially offer the benefits of corticosteroid treatment – ameliorating muscle weakness and improving cardiac function – while minimizing detrimental side effects such as impaired growth and increased susceptibility to infections.

Moreover, this research provides a framework for exploring the cell-autonomous roles of nuclear receptors in muscular dystrophy, paving the way for a deeper understanding of the molecular underpinnings of DMD and other similar neuromuscular disorders. By dissecting the mechanisms through which GR signaling protects muscle and heart tissues, future studies can identify novel drug targets and therapeutic strategies that specifically modulate these pathways, potentially leading to more effective and less harmful treatments for patients with DMD.

**Figure:**
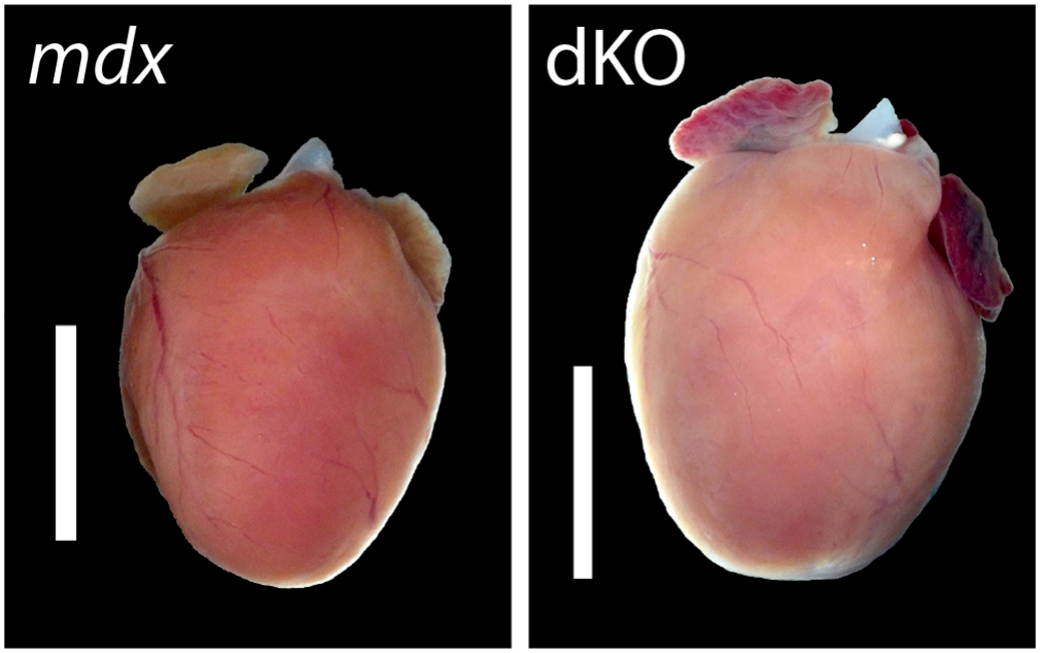
**The glucocorticoid receptor might be indicative of worsening dystrophic cardiomyopathy in Duchenne muscular dystrophy. **In the right image, the double knockout (dKO) mouse model, which lacks both dystrophin and the glucocorticoid receptor, shows a significantly larger heart (by about 18%, *P*<0.01) than that of the *mdx* mouse model, which lacks dystrophin only. Scale bars: 4 mm.

**Why did you choose DMM for your paper?**

We have always enjoyed DMM as a journal for its Open Access approach to science and how it embraces interdisciplinary research. For this particular paper, we are excited that DMM allows us to reach beyond some of the more neuromuscular disease- or muscle-specific journals to reach a broader scientific community. Considering that glucocorticoids are used to treat hundreds of different indications, we think that diverse groups of DMM readers will be excited by our work, which establishes a new disease model that can be used to test mechanisms of this pathway. Additionally, we appreciate what we have seen regarding the philosophies of this journal, including its thorough approach to ensuring scientific rigor, as well as its biodiversity initiative, through which DMM plants a tree for every Research Article published.

**Given your current role, what challenges do you face and what changes could improve the professional lives of other scientists in this role?**

Navigating the interdisciplinary landscape of the biomedical and translational sciences, particularly for complex conditions like DMD, presents the dual challenges of mastering diverse methodologies and overcoming academic silos. To mitigate these challenges, a strong emphasis on promoting a culture of open science is essential, where sharing data, methods and findings openly accelerates research and fosters collaboration. Additionally, recognizing and valuing interdisciplinary work within academic evaluations and funding decisions is crucial for encouraging innovative research approaches. Institutions should adapt their frameworks to better appreciate the nuanced contributions of interdisciplinary efforts, which often drive significant scientific breakthroughs. By enhancing support structures, such as offering interdisciplinary training and facilitating cross-disciplinary dialogue, researchers can more effectively combine diverse expertise towards solving complex health issues. Embracing open science principles and acknowledging the importance of interdisciplinary research will not only advance our understanding of diseases like DMD but also pave the way for novel therapeutic interventions. Ultimately, these shifts will enrich the scientific community, fostering an environment where collaborative innovation thrives.

**What's next for you?**

Now as a first-year PhD student at Cedars-Sinai Medical Center, I want to continue studying how sex and genetics may lead to distinct phenotypes in the immune system. As researchers, we have access to an overwhelming amount of publicly available datasets, which helps us generate baseline hypotheses from pretty much any interest. I am particularly interested in studying how we can use information to enhance precision medicine in diverse patient populations and see immense value in supplementing wet-lab experiments with computational methods to maximize results without unnecessarily repeating studies. However, my end goal is to become an expert in my research so that I can effectively communicate major findings with a general audience.

**Tell us something interesting about yourself that wouldn't be on your CV**

As a future biomedical scientist, I realize how important diet is to health, which is why I love spending my weekends finding the best local produce at farmers markets! Seriously, if you tasted an in-season strawberry just a few hours after it was harvested, I don't think you'd ever shop at another grocery store for fresh foods. I used to be an urban farmer in Washington DC and I hope to have my own hobby farm in the future.
